# Polarized vision in the eyes of the most effective predators: dragonflies and damselflies (Odonata)

**DOI:** 10.1007/s00114-025-01959-3

**Published:** 2025-01-21

**Authors:** Rodrigo Roucourt Cezário, Vinicius Marques Lopez, Felipe Datto-Liberato, Seth M. Bybee, Stanislav Gorb, Rhainer Guillermo-Ferreira

**Affiliations:** 1 LESTES, Entomology and Experimental Biology Center, Federal University of Triângulo Mineiro (UFTM), Uberaba, MG Brazil; 2 Graduate Program in Entomology, University of São Paulo (USP), Ribeirão Preto, SP Brazil; 3 Guajará-Mirim Integrated Management Nucleus, Chico Mendes Institute for Biodiversity Conservation (ICMBio), Guajará-Mirim, RO Brazil; 4 Department of Biology and Monte L. Bean Museum, Brigham Young University, Provo, UT 84602 USA; 5 Department of Functional Morphology and Biomechanics, Zoological Institute, Kiel University, Kiel, Germany

**Keywords:** Optics, Morphology, Physiology, Insect vision, Eyes

## Abstract

Polarization is a property of light that describes the oscillation of the electric field vector. Polarized light can be detected by many invertebrate animals, and this visual channel is widely used in nature. Insects rely on light polarization for various purposes, such as water detection, improving contrast, breaking camouflage, navigation, and signaling during mating. Dragonflies and damselflies (Odonata) are highly visual insects with polarization sensitivity for water detection and likely also navigation. Thus, odonates can serve as ideal models for investigating the ecology and evolution of polarized light perception. We provide an overview of the current state of knowledge concerning polarized light sensitivity in these insects. Specifically, we review recent findings related to the ecological, morphological, and physiological causes that enable these insects to perceive polarized light and discuss the optical properties responsible for the reflection of polarized light by their bodies and wings. Finally, we identify gaps in the current research and suggest future directions that can help to further advance our knowledge of polarization sensitivity in odonates.

## Introduction

Polarized light is abundant in nature. It occurs when light waves produced by the sun are reflected or scattered in the environment. Insects use polarized light as cues for a wide range of visual tasks, such as celestial navigation (Wehner and Müller 2006; Homberg et al. 2011; Mathejczyk and Wernet 2019), contrast enhancement (Sharkey et al. 2015; Marshall et al. 2019), water detection (Wildermuth 1998), host detection, and sexual signaling (Yadav and Shein-Idelson 2021). Polarization sensitivity can, however, interfere with color and motion detection systems, for example, by inducing false color artifacts (Kelber et al. 2001). Some species have, therefore, evolved adaptations that reduce sensitivity to polarized light, such as photoreceptor twist (Wehner and Bernard 1993). By contrast, some insects, such as butterflies, appear to have some degree of polarization sensitivity in all photoreceptor cells within the main retina (Kinoshita and Arikawa 2014).

**Fig. 1:**
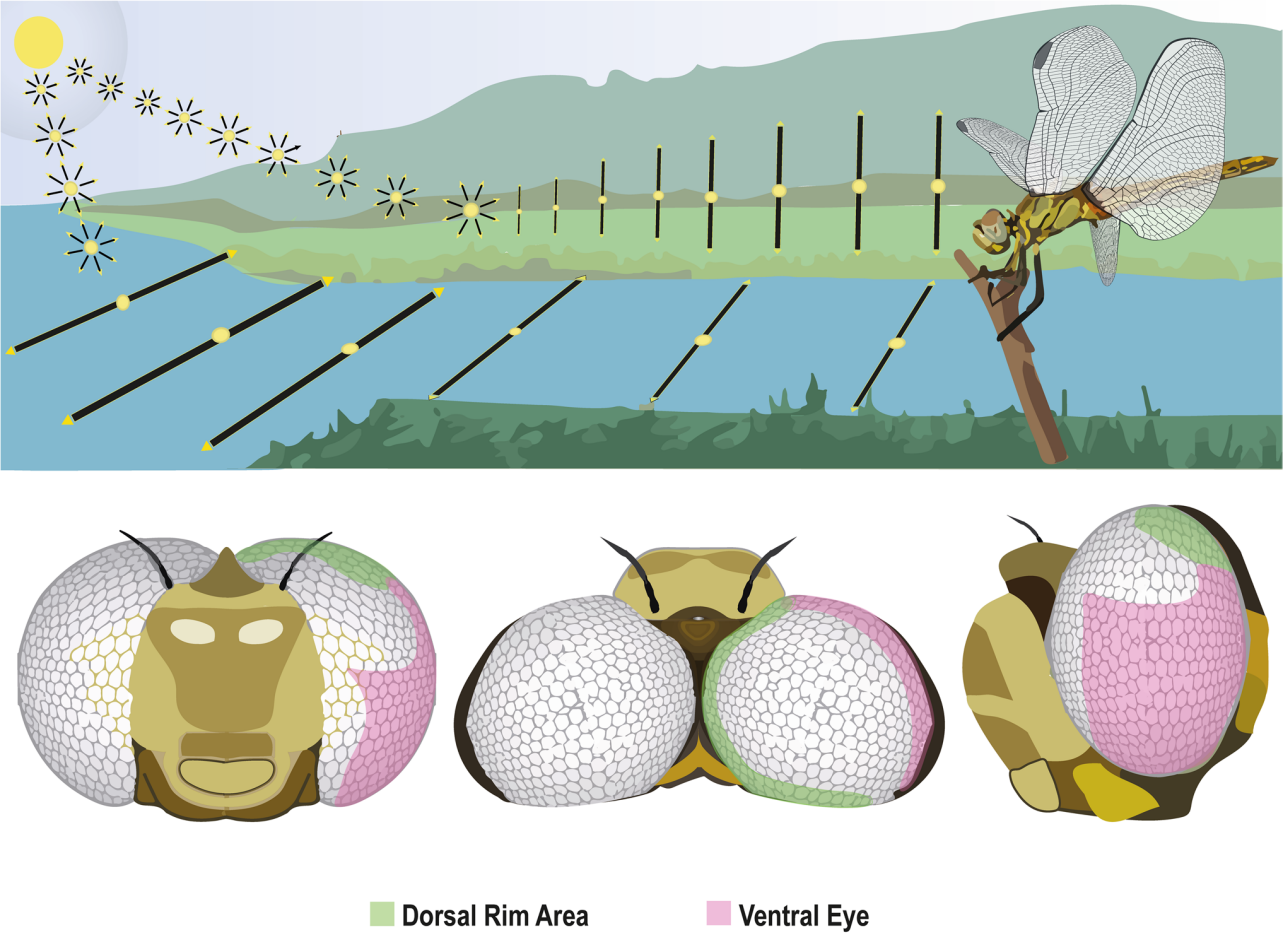
Specialized ommatidia sensitive to polarization can be found in odonates’ dorsal rim area (DRA; green) and ventral eye (purple) regions (adapted from Lancer et al. 2020). Unfilled eye regions indicate areas where evidence for the presence of polarization-sensitive cells is insufficient. When perched near water—rendezvous and oviposition sites for odonates—the skyward-facing DRA detects skylight polarization patterns, while the ventrally oriented polarization-sensitive cells detect polarization reflected from water or the ground

Many navigating insects, including odonates, possess a specialized polarization-sensitive region of the eye called the dorsal rim area (DRA) (Meyer and Labhart 1993; Labhart and Meyer 1999). The skyward-facing DRA functions to detect skylight polarization patterns that are formed by light scattering in the atmosphere. Ventrally oriented polarization-sensitive cells enable the detection of water- or ground-reflected polarization (Meinertzhagen et al. 1983; Schwind 1991) and polarization signals due to reflection from the cuticle of conspecifics (Fig. 1). Deeper investigation into the role of polarization detection plays in mediating insect behavior is needed, but it is likely to be particularly interesting within specific groups of insects, such as the odonates.

Dragonflies and damselflies are important insect taxa in vision research, as many aspects of their natural history are strongly influenced by their visual capabilities. Nearly all odonates live most of their life cycle in aquatic environments but become terrestrial as adults, where they are unmatched flyers and are primarily guided by vision (Corbet 1999). The transition from aquatic immature stages to terrestrial adults provides an opportunity to explore visual adaptations to different light environments within a single species and across odonate diversity.

As such, odonates provide an excellent model for exploring the visual ecology and evolution of polarization vision and its associated adaptational mechanisms (Wildermuth 1998; Kriska et al. 2006, 2009; Horváth et al. 2007; Bybee et al. 2012, 2022; Sharkey et al. 2015; Ensaldo-Cárdenas et al. 2021). Further, several groups of odonates have terrestrial or semi-terrestrial immature stages, which makes for additional interesting avenues for research (Corbet 1999). The role of polarized light as a source of information during habitat selection, communication, and navigation is not well understood in damselflies or dragonflies.

Modern Odonata represents the extant members of an ancient insect order and is currently hypothesized to be a sister (along with mayflies) to all winged insects (Misof et al. 2014). Thus, Odonata provides valuable models for ecological and evolutionary studies that can yield insights into the evolutionary processes that have led to the diverse morphological specializations and physiological mechanisms related to polarization vision found across Insecta (Bybee et al. 2016; Córdoba-Aguilar et al. 2023).

The following sections provide a comprehensive overview of the biological significance of polarized light perception in Odonata, based on the available evidence we reviewed. We focus on their morphological specializations, physiological mechanisms, polarization-mediated behaviors, and the optical properties underlying reflected polarized light. Specifically, we aim to shed light on (i) the current state of knowledge surrounding polarization-mediated habitat selection, celestial navigation, and intra- and interspecific communication in odonates; (ii) the morphological specializations and physiological mechanisms by which these insects perceive polarized light; (iii) the optical properties behind reflected polarized light from odonate cuticle and/or wings; and (iv) identify gaps in the current knowledge and suggest future research directions. By advancing our understanding of the role of polarized light in odonate behavior, we hope to contribute to a broader understanding of animal perception and ecological adaptations.

### What is polarized light?

**Fig. 2:**
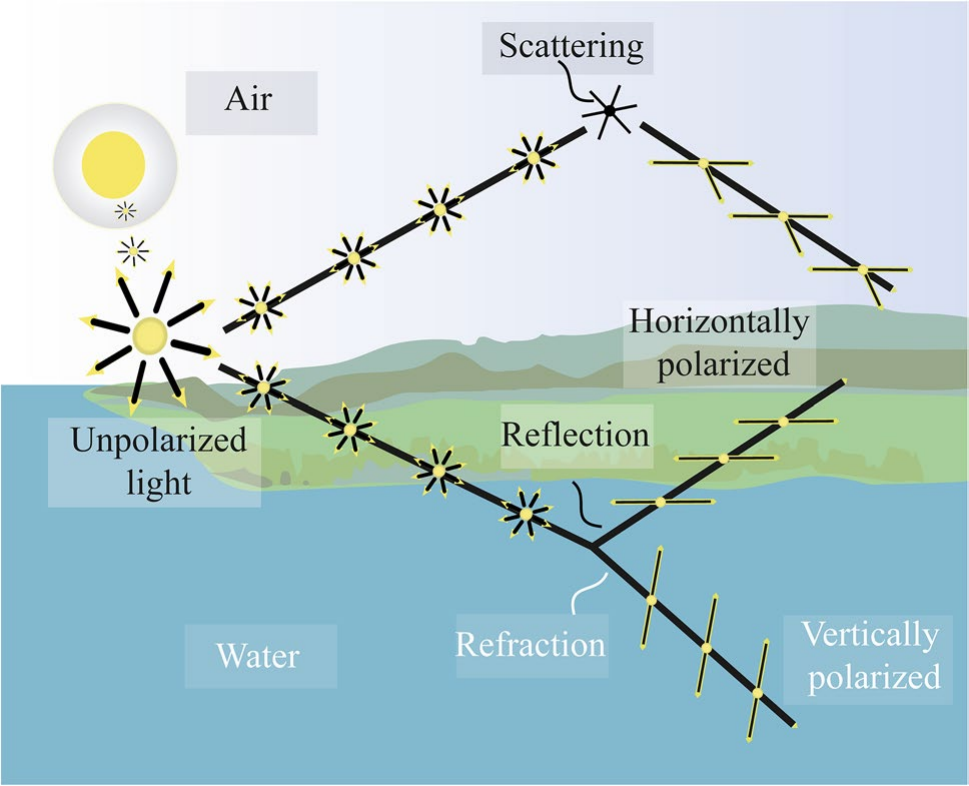
Light polarization in nature. Unpolarized sunlight becomes linearly polarized, either vertically or horizontally, through reflection from surfaces, refraction upon entering matter, or scattering by particles smaller than one-tenth of its wavelength. Due to the specialized mechanisms required for producing and detecting circularly polarized light, it will not be explored further here

Polarization is a property of light that describes the oscillation of the electric field vector (e-vector) that lies in a plane orthogonal to the direction of propagation (Goldstein 2017). The predominant angle of e-vector orientation describes the angle of polarization, and the degree to which this orientation occurs (from 0 unpolarized light to 1 completely linearly polarized light) describes the degree or percent polarization (DoP) (Konnen 1985). Polarized light is formed when unpolarized sunlight interacts with the environment, through scattering, reflection, or refraction, in a way that the e-vectors of that light become oriented to one plane (DoP = 1); that light has become linearly polarized (Fig. 2). Linearly polarized light can be horizontally polarized, vertically polarized, or polarized at an angle between the two (e.g., the rainbow is tangentially polarized) (Konnen 1985; Goldstein 2017). In nature, light is commonly partially polarized (DoP > 0) (Marshall and Cronin 2011; Goldstein 2017). Polarized light may also exhibit ellipticity, or become circularly polarized, whereby the e-vector angle rotates with the propagation of the light (Goldstein 2017).

### Where is polarized light found in nature?

Light polarization is a common phenomenon in nature with three primary sources: scattering from sub-wavelength atmospheric molecules, such as nitrogen and oxygen or water molecules (known as Rayleigh scattering), and reflection from dielectric surfaces like mud, bodies of water, or arthropod cuticle (Horváth 1995; Lerner 2014; Goldstein 2017). Polarization may be found in skylight, leaves, soil, water, and other smooth surfaces (Waterman 1954; Horváth 1995; Lerner 2014; Goldstein 2017), as well as unconventional sources such as bark resin, crude oil, automotive clearcoats, and gravestones, which may pose ecological traps for insects due to their polarotactic behavior (Watson 1992; Wildermuth 1992; Horváth et al. 1998, 2007, 2021; Stevani et al. 2000a, b; Bernáth et al. 2001; Burrial and Ocharan 2003; Wildermuth and Horvéth 2005).

**Fig. 3:**
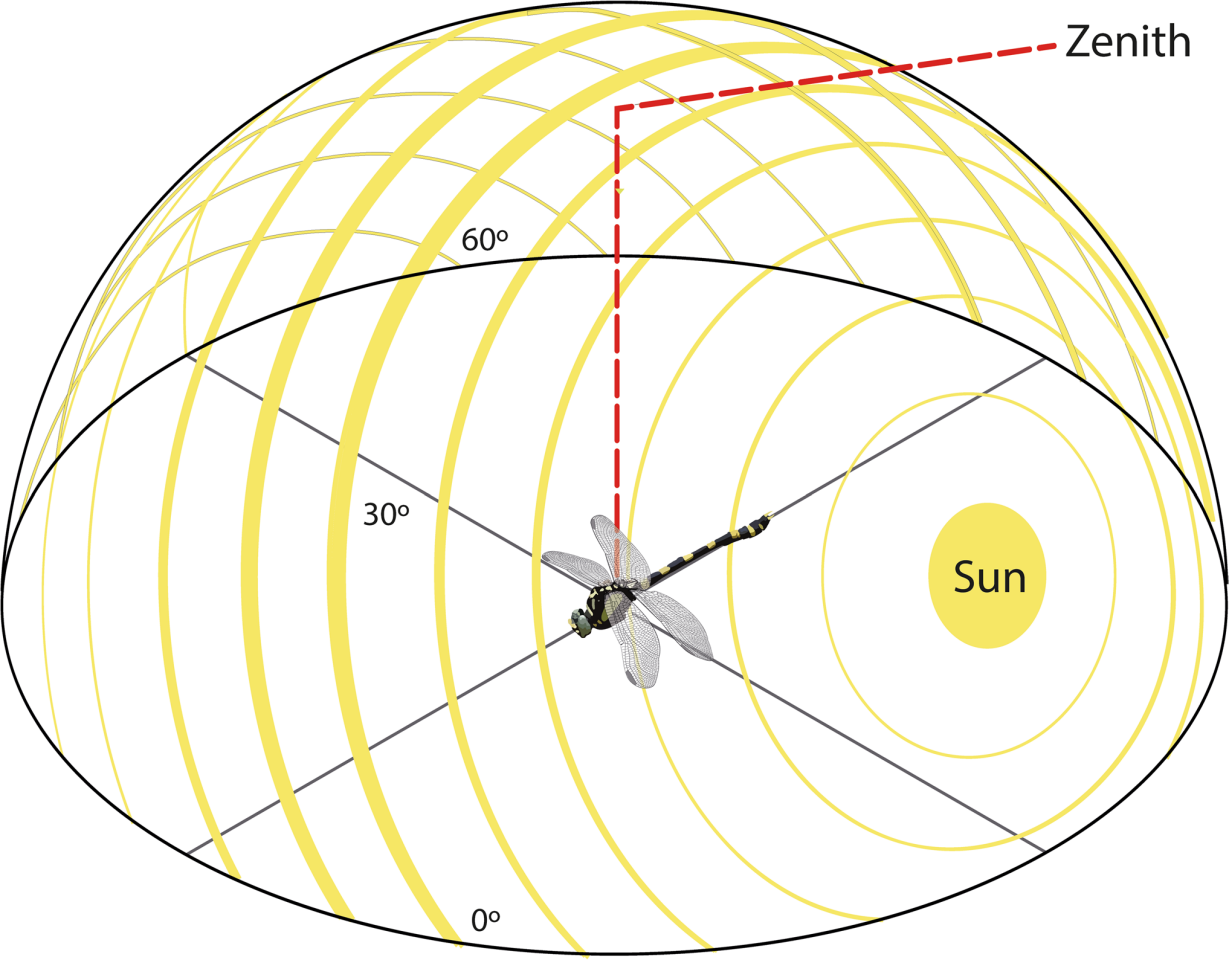
Illustrative diagram of polarized light patterns in the sky. The direction of the yellow lines depicts the e-vector direction of propagation, and their thickness indicates the degree of polarization. Maximum polarization occurs 90° from the sun, while light near the sun and antisun remains unpolarized. The zenith, representing the imaginary point intercepted by a vertical axis extending from the observer’s head to the celestial sphere, is also indicated. Adapted from Heinze (2014)

Polarized light from Rayleigh scattering in the sky forms predictable e-vector patterns that some animals use for navigation. This pattern consists of concentric circles around the sun, with the highest polarization occurring 90° away from it, while light near the sun and the “antisun” (on the opposite side of the sky) is unpolarized (Heinze 2014) (Fig. 3). Because the e-vector pattern is directly linked to the sun’s position and shifts as the sun moves across the sky, animals can determine solar location even when only a small patch of blue sky is visible, aiding in navigation and orientation (Wehner and Müller 2006; Heinze 2014).

Flying insects are constantly exposed to various sources of linearly polarized light, which can serve as important sources of information for navigation, communication, water seeking, and prey detection (Yadav and Shein-Idelson 2021; Johnsen 2012). Underwater, light polarization is influenced by several factors, including the angle of the sun, which affects the angle and degree of polarization (Cronin and Shashar 2001; Waterman 2005). As sunlight penetrates the water surface, it becomes partially polarized, and the DoP is influenced by scattering from water molecules and larger particles, such as plankton and detritus (Horváth and Varjú 1997; Marshall and Cronin 2011; Horváth 2014). The polarization pattern underwater can change with the time of day, water clarity, and the presence of particulates, making it a dynamic environment for polarization-sensitive organisms. Polarization sensitivity in odonate larvae is known to increase their contrast detection in the partially polarized underwater light environment they inhabit, enhancing their ability to detect prey and navigate effectively (Sharkey et al. 2015).

Circularly polarized light, whether clockwise or counterclockwise, is determined by the chirality of the reflective molecules. This intriguing phenomenon occurs in various organisms and natural surfaces (Neville and Caveney 1969; Wynberg et al. 1980; Hegedüs et al. 2006; Sharma et al. 2009; Johnsen 2012; Vignolini et al. 2012). A particularly remarkable example is the mantis shrimp (*Gonodactylaceus falcatus* (Forskål)), which is unique in its ability to detect both clockwise and counterclockwise circularly polarized light as well as linearly polarized light, thanks to specialized optical mechanisms involving quarter wavelength retarders (Chiou et al. 2008; Gagnon et al. 2015). While circularly polarized light has been reported in some beetles and other organisms (Wynberg et al. 1980; Vukusic et al. 2004; Sharma et al. 2009; Baar et al. 2014), the evidence for its detection and biological significance in these cases is scant (Brady and Cummings 2010; Blahó et al. 2012). Circularly polarized light can occur underwater due to internal reflection and scatter of linearly polarized light, suggesting potential navigational uses for aquatic animals (Cronin et al. 2014).

It remains unknown whether dragonflies and damselflies can detect or distinguish between different types of circularly polarized light. Given the specialized mechanisms required to perceive such light along with our limited understanding of these processes, we will not explore this topic in greater depth here. This area of research holds potential for future studies, which could uncover new insights into the evolutionary and ecological roles of circularly polarized light in nature.

## How do odonates detect polarized light?

Insects can perceive polarization due to the biochemical and biophysical properties of the retinal chromophore, a derivative of vitamin A essential for vision in all animals. The chromophore binds to an opsin protein, forming the visual pigment that initiates vision by absorbing photons. The probability of light absorption increases when the e-vector of light is parallel to the chromophore, making the process more efficient. Visual pigments are arranged in cylindrical microvilli within the rhabdom, and their preferential orientation with the microvillar axis is understood to result in polarization sensitivity (Roberts et al. 2011; Johnsen 2012; Cronin et al. 2014). Therefore, light is maximally absorbed when photoreceptor microvilli are aligned with the angle of the incoming polarized light and reduced to a minimum when light is polarized at 90° to this angle. A multi-channel detector system has the potential to analyze the degree and angle of polarization via downstream neural processing. Insect polarization detectors feature photoreceptors with microvilli oriented in multiple orientations (e.g., orthogonally) within or across ommatidial units.

Odonates are visually oriented insects with remarkable color (Futahashi et al. 2015; Suvorov et al. 2017) and polarization sensitivity (Kriska et al. 2009; Sharkey et al. 2015). Their compound eyes, composed of up to 30,000 individual ommatidia in the adult eyes, allow them to detect sources of light polarization in their natural habitats. The first demonstration of polarization sensitivity in this taxon showed that retinula units change their electrical response according to the angle of light polarization (Horridge 1969).

Polarization-sensitive photoreceptors are found in the frontal and ventral regions of the eyes (Laughlin 1976a; Meinertzhagen et al. 1983; Brydegaard et al. 2018) (see also Labhart and Meyer 1999; Laughlin 1976b; Meyer and Labhart 1993; Snyder et al. 1973). For instance, *Hemicordulia tau* Selys has UV and blue retinula cells in their ventral retina with high sensitivity to light polarization (Laughlin 1976b; Laughlin and McGinness 1978). UV-sensitive receptors have orthogonally oriented vertical and horizontal microvilli across different ommatidial units, likely serving as horizon and/or water detectors to aid in stability during flight and the detection of mating and/or oviposition sites.

Dragonflies, like many other navigating insects, have specialized photoreceptors in the dorsal rim area (DRA) of their compound eyes, likely serving as polarization detectors (Meyer and Labhart 1993). Ommatidia from DRA are characterized by short rhabdoms, minimizing self-screening, and lack of a rhabdomeric twist. They consist of seven retinular cells (instead of the typical eight found in other eye regions) that extend through the full length of the ommatidia and exhibit rudimentary corneal lenses and reduced crystalline cones (Horridge 1969; Meyer and Labhart 1993; Labhart and Meyer 1999). Although it is likely that the DRA is used for odonate navigation, this is yet to be tested behaviorally.

As amphibiotic insects, damselflies and dragonflies spend much of their time in and around water, relying primarily on vision for navigation and prey detection (Corbet 1999). Their polarization sensitivity, which likely evolved early in their evolutionary history, enables them to perceive patterns of polarized light invisible to humans and other animals lacking this ability (Meyer and Labhart 1993; Horváth and Varjú 2004). This sensitivity may play a substantial role in behaviors such as mate recognition, predator avoidance, and dispersion (Meyer and Labhart 1993; Horváth and Varjú 2004; Csabai et al. 2006; Sharkey et al. 2015). Additionally, the polarization of light reflecting off water surfaces provides important navigational information, indicating the location and orientation of water bodies (Wildermuth 1992; Horváth and Varjú 2004; Ensaldo-Cárdenas et al. 2021). This ability gives damselflies and dragonflies a significant advantage in their aquatic and terrestrial environments.

## Summary of evidence on the use of polarized light in odonate behavior

**Fig. 4:**
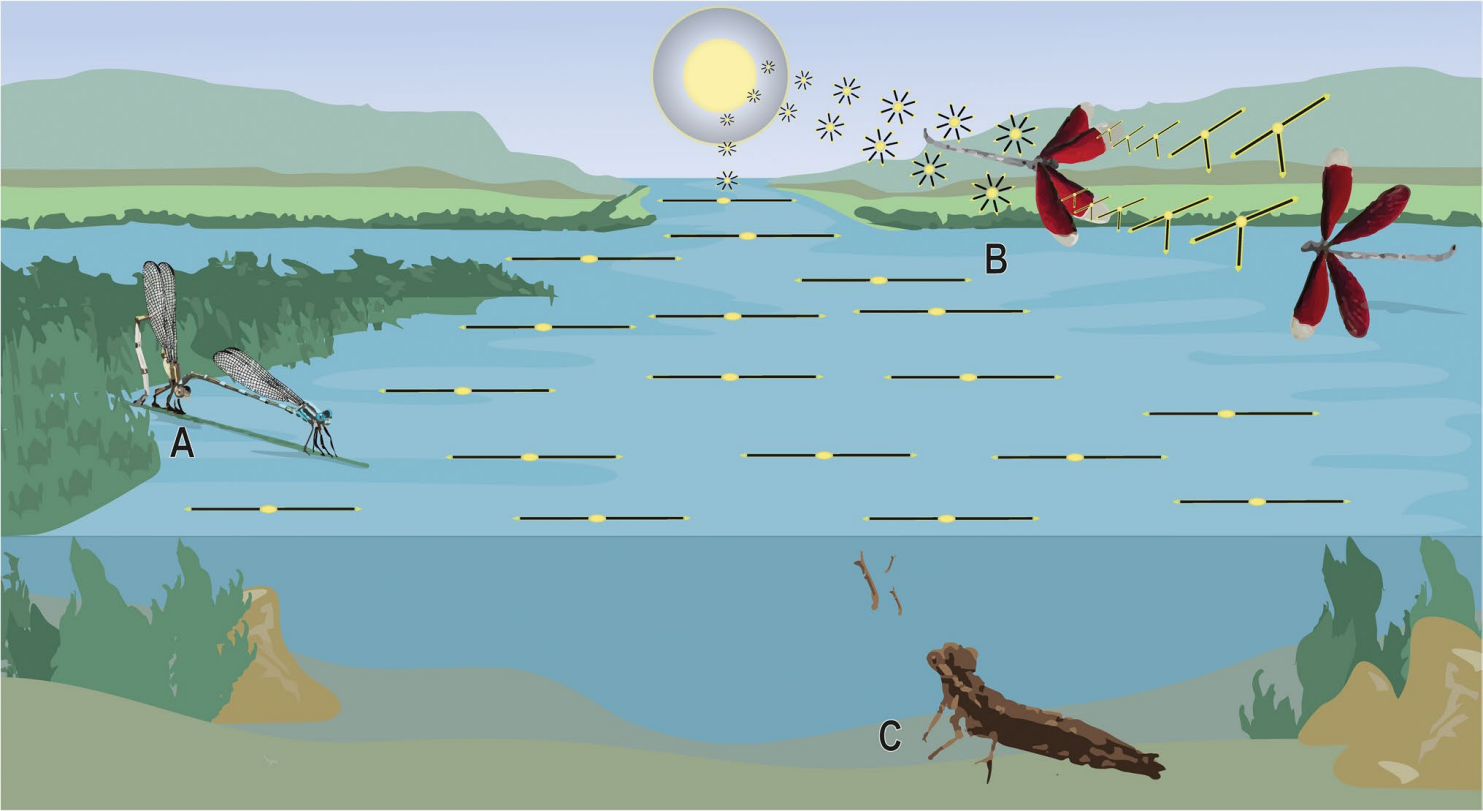
Proposed and hypothetical primary uses of polarized light by odonates. The insects may use light polarization in behavioral situations as follows. **A** To select suitable oviposition and/or rendezvous sites. **B** During agonistic flights to communicate their resource-holding potential or to enhance the contrast of their body colors. **C** To detect prey underwater (and hypothetically, against brightly contrasting backgrounds, such as the blue sky during flight)

The ability of odonates to detect polarized light may allow them to navigate their environments, hunt for prey with incredible precision, and aid in intra- and interspecific communication. Here, we summarize the ways odonates might use polarized light (Fig. 4).

### Habitat selection

Most studies addressing the role of polarized light in odonate natural history focus on habitat selection (Fig. 4(A)). For instance, adult odonates are attracted to water surfaces that reflect horizontally polarized light and can distinguish between vertically and horizontally polarized light to identify suitable habitats (Horváth 1995; Schwind 1995; Wildermuth 1998; Bernáth et al. 2002; Horváth and Varjú 2004). They are capable of distinguishing between ultraviolet and non-ultraviolet polarized reflecting water bodies. Some may even prefer those that reflect UV (Ensaldo-Cárdenas et al. 2021), aligning with the UV- and polarization-sensitive photoreceptors that have been found in the ventral region of *H. tau*’s eyes (Laughlin 1976b; Laughlin and McGinness 1978). UV stimuli may aid odonates to identify rendezvous locations (Wildermuth 1998) and mate recognition (Guillermo-Ferreira et al. 2014). Thus, a hypothesis may be that males and females use these information (e.g., UV-polarization) to find territories and mating and/or oviposition sites (Ensaldo-Cárdenas et al. 2021).

### Intra and interspecific communication

It is well known that odonates use visual signals for communication. For instance, bright colors may be used during male-male contests (Guillermo-Ferreira et al. 2015a, b, 2019), or to recognize (Guillermo-Ferreira et al. 2014) and to attract mates (Pena-Firme and Guillermo-Ferreira 2020), or even hide from predators and prey alike (Cezário et al. 2021, 2022a, b). It is possible that polarized light may play a role in these intra- and interspecific communication channels, although further research is needed to test this hypothesis.

The body and wings of several adult odonates have the potential to alter the polarization of reflected light (Fig. 4(B)). The reflection of light polarization in Odonata is known from the colored hindwings of the Phoenix damselfly *Pseudolestes mirabilis* Kirby (Zygoptera: Pseudolestidae) (Nixon et al. 2017) and from the hyaline wings of the Blue Hawker *Aeshna cyanea* (Müller) (Anisoptera: Aeshnidae) (Hooper et al. 2006).

The wings of the Blue Hawker, like most other dragonflies, are transparent and covered with wax-derived pruinosity, but with no detectable birefringence (Newman and Wootton 1986; Gorb 1995). Hooper et al. (2006) reported “leaky” guided modes in the transparent wings of *A. cyanea*, which result in the reflectance of partially polarized light by as much as 30% at incidence angles around 45° and up to 60% at much higher angles. For instance, females may observe the orientation of male wings and their degree of polarization (Hooper et al. 2006; Brydegaard et al. 2018). The reflected polarized light from male wings or body may provide important signals of male reproductive potential, similar to what has been observed in *Heliconius* butterflies (Sweeney et al. 2003). As a result, structures that reflect polarized light may be subject to sexual selective pressures.

For *P. mirabilis*, Nixon et al. (2017) reported a remarkable case of structural coloration in their hindwings. The ventral surface of the hindwings has a bright-white patch caused by an epicuticular wax secretion arranged in flattened parallel fibers, which forms a two-dimensional scattering structure that reflects linear polarized light. The average reflectance of the white patch can reach up to 58% for polarized light when the e-vector axis is parallel to the wax fibers and 47% when the e-vector axis is perpendicular (Nixon et al. 2017). The copper–gold dorsal surface of *P. mirabilis* males also reflects linearly polarized light, though in less intensity than that of the white patch. Light polarization may be more widespread among Odonata, but additional focused research is needed to establish just how common, or rare, it may be.

### Prey detection

Polarization sensitivity has been observed in the larvae of the emperor dragonfly, *Anax imperator* Leach in Brewster, as an adaptation for contrast enhancement (Sharkey et al. 2015) (Fig. 4(C)). This adaptation reduces the polarized scatter underwater, increasing the visibility of viewed objects. To date, no studies have investigated polarization sensitivity and prey detection in adult dragonflies, even though they have spectral adaptations to detect prey against the blue sky (Labhart and Nilsson 1995).

The fiddler crab, *Uca stenodactylus* Edwards & Lucas (How et al. 2015), and some cephalopods, such as cuttlefish and squids, also use polarization contrast for target detection in their natural environments. Blood-feeding tabanid flies are less attracted to white than to dark-colored horses, since they use polarized light reflected from the coat as a cue to locate a host (Horváth et al. 2010). This preference for black and brown fur is attributed to positive polarotaxis (Horváth et al. 2010). These findings suggest that polarization sensitivity may be widespread in insects and an important adaptation for detecting targets in a variety of natural environments (Shashar et al. 1998, 2004; How et al. 2015; Venables et al. 2022). Adult odonates have a complex system of prey detection and tracking against the sky (Olberg et al. 2000), which may also be aided by polarized sensitivity.

## Polarized vision and environmental traps of dragonflies

**Fig. 5:**
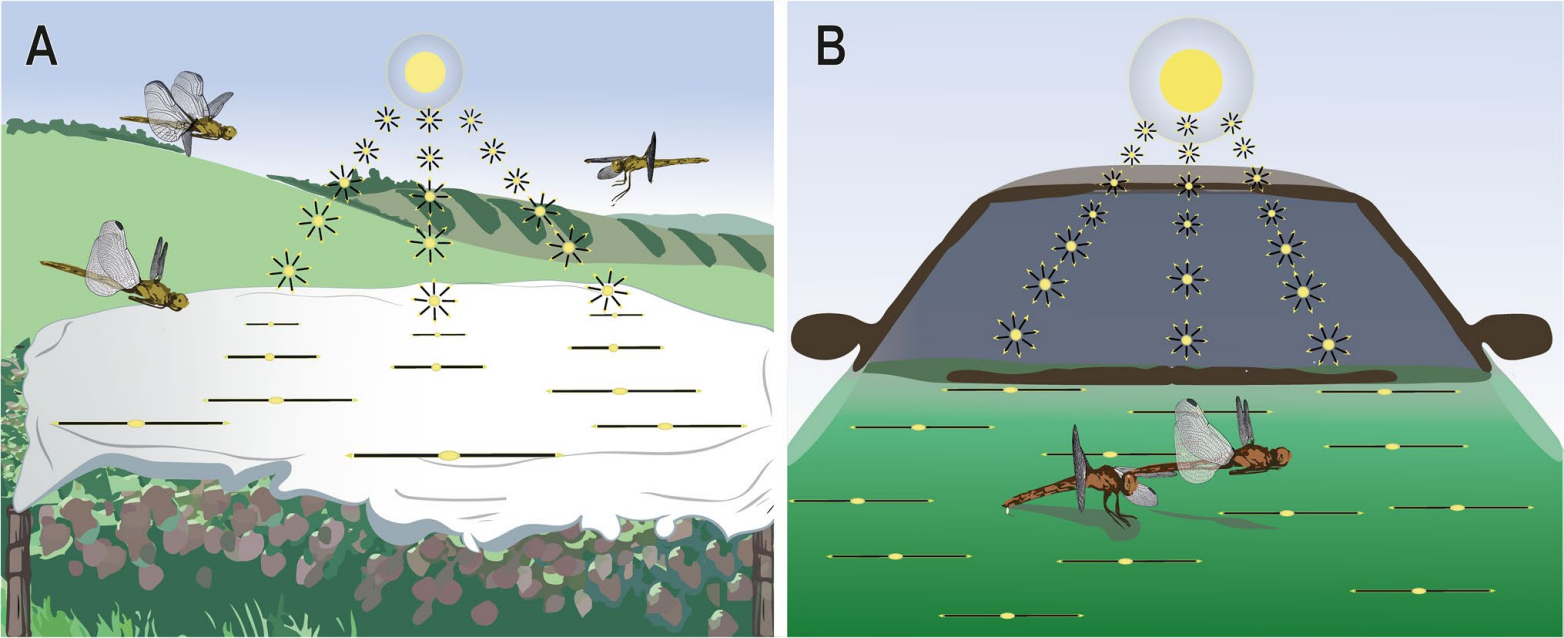
Polarotactic reactions of dragonflies to environmental traps.** A** Dragonflies are attracted to polarized light reflected by plastic sheeting, which can lead to failed reproductive attempts.** B** Similarly, polarized reflections from car surfaces may also act as environmental traps

Polarotactic reactions of dragonflies to plastic sheeting, car surfaces, and other smooth surfaces have been previously described (Watson 1992; Wildermuth 1992; Stevani et al. 2000b, a; Bernáth et al. 2001; Burrial and Ocharan 2003; Wildermuth and Horvéth 2005; Horváth et al. 2007) (Fig. 5). Such polarized artificial surfaces attract reproductively active individuals, perhaps at high population densities. In northeastern Switzerland, *Coenagrion puella* (L.) (Coenagrionidae) and *Libellula quadrimaculata* L. (Libellulidae) were observed in numbers away from water bodies in the strawberry fields covered by shiny black plastic sheets (Wildermuth 2007) (Fig. 5A). The dragonflies likely mistook the plastic sheeting for a body of water since the surface of plastic sheeting likely produces polarized light similar to that reflected from water surfaces (Wildermuth 1992). Both sexes exhibited typical elements of the species-specific reproductive behavior around the sheeting, including oviposition attempts. Tandem pairs of *Sympetrum vulgatum* (L.) (Libellulidae) were observed ovipositing eggs onto the metallic-green bonnet of a car parked in the sun (Günther 2003) (Fig. 5B). Abnormal egg-laying sites on polarized glass surfaces have also been observed in *Libellula depressa* L. (Wyniger 1955) and *Sympetrum striolatum* (L.) (Paine 1992). *Sympetrum striolatum* has also been observed ovipositing on the plastic windshield of the car (Paine 1992). Since dragonflies cannot distinguish the polarized light of plastic sheeting and car surfaces from natural water bodies, they expend unnecessary energy and genetic reproductive material (i.e., sperm and egg). Due to their erroneous habitat choices, one may consider such human-made surfaces along with some light sources as ecological traps (Wildermuth 2007; Fraleigh et al. 2021). However, since there are few such observations from the literature, the negative effect of such surfaces on dragonfly populations in a human-modified landscape is unknown and probably negligible.

## Research gaps

The study of visual signals in odonate communication has gained increasing attention in recent research (Suárez‐Tovar et al. 2022). Nevertheless, despite its potential importance, the role of polarization signals in intraspecific communication within Odonata remains largely unexplored, with little empirical evidence to date. However, as seen in other insect taxa, such as the Asian swallowtail *Papilio xuthus* L. (Papilionidae), polarization contrast may be used to detect motion during foraging and mate-seeking (Stewart et al. 2015, 2019). Considering the complex color-producing mechanisms used by odonates for intra- and interspecific communication (Guillermo-Ferreira et al. 2015a; Cezário et al. 2022a, b), polarization sensitivity may also play an important role in odonate behavior.

The complex life cycle of these animals, which involves both underwater and terrestrial phases, makes it possible, if not likely, that they use polarization sensitivity in different aspects of their natural history. Further, because they are easily trackable with mark and recapture experiments in nature (Córdoba-Aguilar et al. 2023) and in the lab (for larvae), the possibility to test hypotheses related to polarization sensitivity is possible. Odonates exhibit a range of complex behaviors that depend on their visual abilities and access to information from their environment, whether to find suitable oviposition sites or to display their body colors for mates and/or rivals to detect. Some species are known to undertake long-distance migrations across entire continents, similar to migratory songbirds, and even across large areas of open ocean at certain times of year (Wikelski et al. 2006; Anderson 2009). Successively low temperatures and wind currents play a major role in odonate navigation, but polarization may also play a part. There is currently no evidence for polarization-based celestial orientation in odonates. Investigating this topic may yield new insights into their behavior and adaptations.

The underwater environment presents unique challenges for visual perception, such as the scattering of light in water, which can affect the perception of polarized light (Chiou et al. 2008). The role of polarization sensitivity in the ecology of odonates and their larvae in aquatic environments is therefore an important area of research. In aquatic environments, dragonfly larval polarization sensitivity improves the contrast of the visual scene, assisting in the detection of prey and predators (Sharkey et al. 2015). Understanding the developmental changes in polarization sensitivity (between larvae and adults, or between the different instars of larvae) may shed light on the role of polarization sensitivity in different life stages and the potential trade-offs or constraints associated with it. The expression patterns of opsin genes vary between the different stages of the Odonata life cycle (Bybee et al. 2012; Futahashi et al. 2015). During larval development, several changes occur in their eyes, with the eyes of the adult showing drastic differences from the larva (Sherk 1977, 1978a, b). Some species completely replace the larval eye, while there are some that retain their larval tissue for use in the adult eye (Sherk 1978c). Overall, further research on the role of polarization sensitivity can offer a deeper understanding of odonate behavior and ecology in both aquatic and terrestrial habitats (Heinloth et al. 2018).

Currently, little information is available in the scientific literature regarding the neural circuits and integration processes used by odonates to encode polarized light (Laughlin 1976b). Investigations into neural coding of skylight polarization information from the dorsal rim area have been conducted in a few model species such as the locust *Schistocerca gregaria* Forsskål (Homberg et al. 2023) and the cricket *Gryllus campestris* L. (Homberg et al. 2011) and *Drosophila melanogaster* Meigen (Hardcastle et al. 2021). While other circuits have been described for flight control and small-target detection (Olberg et al. 2000; Mischiati et al. 2015; Supple et al. 2020), the neural processing of polarization information has not been explored in dragonflies or damselflies. Thus, further studies are needed to uncover the neural mechanisms used by odonates to process and integrate polarized light information. Furthermore, the downstream interaction and integration of spectral and polarization channels is yet unknown. Such studies could provide important insights into the evolution of neural mechanisms for polarized light detection across different insect taxa.
